# Fluticasone Induces Epithelial Injury and Alters Barrier Function in Normal Subjects

**DOI:** 10.4172/2157-7536.1000134

**Published:** 2014-06-11

**Authors:** Ruth E. MacRedmond, Gurpreet K. Singhera, Samuel J. Wadsworth, Susan Attridge, Mohammed Bahzad, Kristy Williams, Harvey O. Coxson, Steven R. White, Delbert R. Dorscheid

**Affiliations:** 1 Centre for Heart Lung Innovation, St. Paul’s Hospital, University of British Columbia, Vancouver, Canada; 2 Department of Medicine, University of Chicago, Chicago, Illinois, USA

**Keywords:** Airway epithelial cell, Asthma, Asthma-medication, corticosteroids

## Abstract

**Objective:**

The airway epithelium has a number of roles pivotal to the pathogenesis of asthma, including provision of a physical and immune barrier to the inhaled environment. Dysregulated injury and repair responses in asthma result in loss of airway epithelial integrity. Inhaled corticosteroids are a corner stone of asthma treatment. While effective in controlling asthma symptoms, they fail to prevent airway remodeling. Direct cytopathic effects on the airway epithelium may contribute to this.

**Methods:**

This study examined the effects of a 4-week treatment regimen of inhaled fluticasone 500 μg twice daily in healthy human subjects. Induced sputum was collected for cell counts and markers of inflammation. Barrier function was examined by diethylenetriaminepentacetic acid (DTPA) clearance measured by nuclear scintillation scan, and albumin concentration in induced sputum.

**Results:**

Steroid exposure resulted in epithelial injury as measured by a significant increase in the number of airway epithelial cells in induced sputum. There was no change in airway inflammation by induced sputum inflammatory cell counts or cytokine levels. Epithelial shedding was associated with an increase in barrier function, as measured by both a decrease in DTPA clearance and decreased albumin in induced sputum. This likely reflects the normal repair response.

**Conclusion:**

Inhaled corticosteroids cause injury to normal airway epithelium. These effects warrant further evaluation in asthma, where the dysregulated repair response may contribute to airway remodeling.

## Introduction

Asthma is a chronic inflammatory disease of the airways characterized by reversible airflow obstruction, airway hyper reactivity and airways remodeling [1–4]. Complex interactions between environmental insults and genetically determined host factors generate abnormal injury/repair responses in the airway, resulting in persistent inflammation, epithelial damage and ultimately in the structural changes of airway remodeling. Previously considered a bystander in what was designated primarily an immunological disorder, the role of the epithelium in the pathogenesis of asthma is increasingly recognized [5]. The bronchial epithelium provides chemical, physical, and immunologic barriers to the inhaled environment [6], and has a critical role in orchestrating the inflammatory response to inhaled allergens and pathogens [4]. Loss of epithelial integrity is a hallmark of asthma [7–10]. The potential mechanisms to explain damaged airway epithelium remain unclear and likely are multifactorial, including repetitive exposure to allergic insults (environmental, infectious, inflammatory agents), inflammatory processes [11], a predisposing epithelial dysfunction [5,12] and sub-optimal control with recommended treatment regimens.

Corticosteroids have been the foundation of the pharmaceutical treatment of asthma since the early 1990’s and are used currently as the first line and initial maintenance treatment (“controller”) in patients with recurrent symptoms [1,13]. Corticosteroids are powerful anti-inflammatory agents with a number of cellular targets which reduce recruitment and activation of inflammatory cells in the airway [14]. The glucocorticoid receptor is expressed in airway epithelium [15], and recent work has demonstrated a wide range of additional mechanisms including regulation of epithelial innate and adaptive immune responses [16]. Inhaled corticosteroids have been demonstrated to improve symptoms, exacerbation frequency, and overall quality of life in most patients with asthma [17,18]. However, corticosteroid treatment does not reliably reverse or prevent airway remodeling [19] or alter the natural history of the disease even when therapy is started in childhood [20]. In addition, there is a substantial subset of asthmatics (10–25%) that are resistant to corticosteroid therapy with ongoing symptoms and airway inflammation [21].

Th2-mediated inflammation, which is responsive to inhaled corticosteroid (ICS) therapy, contributes to the pathogenesis and clinical syndrome of asthma, and may have a role in airway remodeling. Abnormalities in the airway epithelium resulting in dysregulated injury, inflammation and repair are recognized to be equally important [22], however in the context of airway remodeling, the anti-inflammatory benefits of corticosteroids may be offset by direct toxic effects on the airway epithelium. Using a variety of *in vitro* and animal models and clinically relevant doses of corticosteroid, we have previously shown that corticosteroids have adverse effects on airway health, increasing epithelial apoptosis, slowing repair and impairing immune responses to viral and bacterial pathogens [23–30]. These adverse effects on the epithelium may occur in parallel with the beneficial anti-inflammatory effects of corticosteroids in asthma, and thus be masked. By studying the effects of inhaled corticosteroids on healthy adults without asthma/airway inflammation, any adverse effects of corticosteroids on the epithelium will be more evident.

The aim of this study was to examine the effect of a 4 week treatment regimen of inhaled corticosteroids on the airway epithelium in healthy human subjects. Damage to the airway epithelium was measured by number of epithelial cells shed into induced sputum. We used two measures of barrier function, examining both bulk diffusive flows as reflected by albumin concentration in the induced sputum and DTPA clearance, which is thought to be a more sensitive measure of tight junction integrity [31]. Differential cell counts and cytokine levels in the induced sputum were measured to reflect airway inflammation.

## Materials and Methods

### Subjects

Healthy subjects aged 18 years and above with no history of smoking, asthma/allergy or other respiratory condition were recruited by newspaper advertising. Subjects were excluded on the basis of abnormal spirometry at baseline screening. The study was approved by the Research Ethics Board (REB) of University of British Columbia/ Providence Healthcare REB# P01-0095. All subjects gave written consent to participate in the study.

### Study Protocol

**Figure 1:**
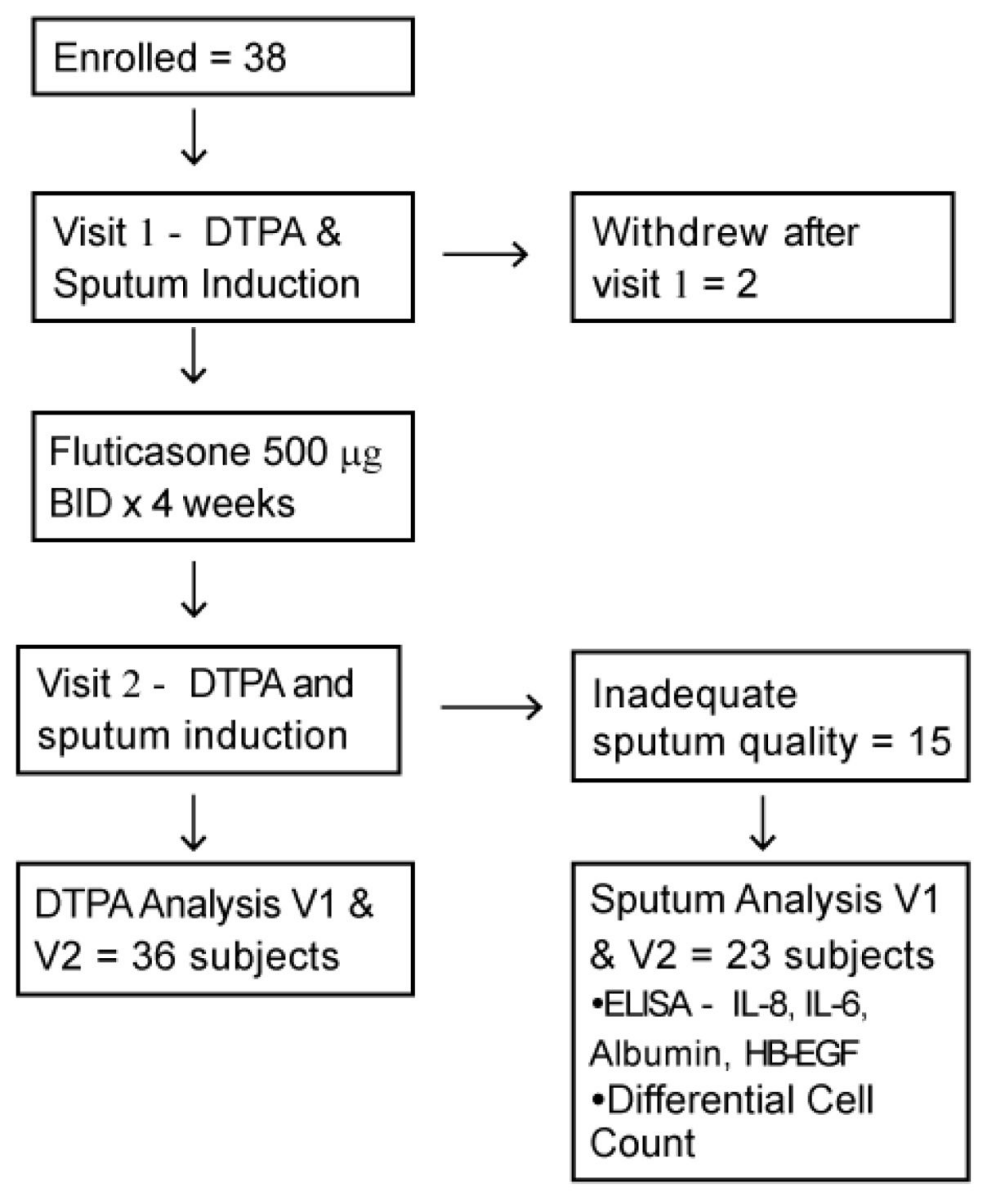
Flow chart of the study protocol. Thirty-six of total thirty-eight normal subjects were given 1000 μg of Fluticasone for four weeks. Samples were collected at visit 1 and visit 2 for DTPA analysis, sputum analysis and differential cell count

This was an uncontrolled before and after observational study (Figure 1). At visit zero [V0] (screening visit), baseline spirometric lung function was obtained in accordance to the standards of the American Thoracic Society (ATS). Only the subjects with an FEV1 of > 80% and with normal lung function were eligible for enrollment into the study. At visit one [V1], a baseline nuclear medicine scintillation scan (DTPA) was performed, and subjects returned the following day for sputum induction. Each subject was then instructed on the proper technique for the delivery of fluticasone via a metered dose inhaler (MDI) with a spacer device. Each subject inhaled fluticasone 250 μg (Flovent, Glaxo SmithKline Inc, Canada) two puffs twice a day (a daily total of 1000 μg daily) for four weeks. Each subject was contacted weekly by the research coordinator to monitor compliance and assess for any adverse events. At visit two [V2] at the conclusion of the inhaled steroid treatment, each subject returned for assessment including symptom review and spirometry and completed a nuclear medicine scintillation scan and a sputum induction following the same protocol as V1.

### 99mTc-DTPA lung clearance test

Transepithelial clearance of DTPA was measured using a nuclear medicine scintillation scan [31]. Subjects inhaled an aeorosolized mist of Technetium-labeled diethylenetriaminepentacetic acid (99mTc-DTPA) via a Fisoneb Ultrasonic nebulizer. Each subject inhaled a dosage of 185 MBq of DTPA for 5 minutes while lying supine on a Siemens PHO/gamma scintillation camera. Subjects were instructed to make rapid inspiratory efforts to ensure central deposition of the particles in the upper airways. Immediately after the delivery of the aerosolized DTPA 30-second image counts were performed for a total of 30 minutes. Anterior and posterior images were taken with a dual headed camera. The region of interest (ROI) was drawn over the central portions of each lung and time-activity curves were derived from the counts per frame computed in the ROI. The T½ value (time required for clearance of 50% of the activity from lung fields) was calculated with the help of the established formula [32]. Decreased T½ denotes faster clearance.

### Sputum induction and processing

Sputum samples were obtained by inhaling increasing concentrations of hypertonic saline solutions (3%, 4%, and 5%) using an ultrasonic nebulizer (Univerisal III model, FLAEM Nuova; Brescia, Italy). The whole sputum sample was collected in a plastic container, weighed, and incubated in 3 volumes of dithiothreitol (DTT) 5 mmol/L (Sputolysin; Calbiochem Corp; San Diego, CA) for 15 minutes at room temperature. An equal volume of phosphate-buffered saline (PBS) was then added to the solution and vortexed for 30 seconds. Cell viability was checked by Trypan blue exclusion. The differential cell count was performed on cytospin products that were stained (Diff-Quick stain; Fisher Scientific; Springfield, NJ). Specimens were considered adequate where viability was >80% and squamous cell contamination was <50%. The bronchial epithelial cells were identified by their columnar shape, round nucleus and cilia on the broad end of the cell. A total of 300 non-squamous cells were counted by two independent blinded observers. Results were averaged and bronchial epithelial cells were expressed as percentage of the total non-squamous cells, and differential counts of inflammatory cells were expressed as a percentage of total inflammatory cells. Following centrifugation at 800 g for 10 min at 4°C, the supernatant was separated from the cell pellet and immediately stored at −80°C for batch analysis.

### Measurement of airway inflammation

The concentrations of inflammatory mediators IL-6 and IL-8 and the repair marker Heparin-binding EGF- like factor (HB-EGF) [33] were measured in induced sputum cell free supernatant in duplicate by standard ELISA. Induced sputum albumin concentration was measured in unprocessed sputum by ELISA and expressed as μg albumin/mg sputum.

### Statistical analysis

Statistical software Graphpad Prism 5 was used for the analysis. Mann-Whitney U test was used for comparison of groups (median values) as the sample size was small and normal distribution could not be assumed. Paired t-test (Wilcoxen) was used to test for effect of treatment between week 0 and week 4. Statistical significance was defined as p-value < 0.05.

## Results

### Study population

Thirty-eight patients were enrolled of which 36 completed the study, two subjects failing to return for the V2 assessment. The 36 consisted of 18 males and 18 females with mean age of 30.9 years (24 – 43) years and average FEV1 of 100 (13.2)% predicted. Paired data for DTPA clearance are presented for all 36 patients.

### Epithelial cell shedding

Sputum induction was of insufficient quality (poor viability or squamous cell contamination) at one or both visits for 13 of 36 patients, thus data for epithelial cell shedding, airway inflammation and albumin influx are presented for 23 subjects (Figure 1).

**Figure 2:**
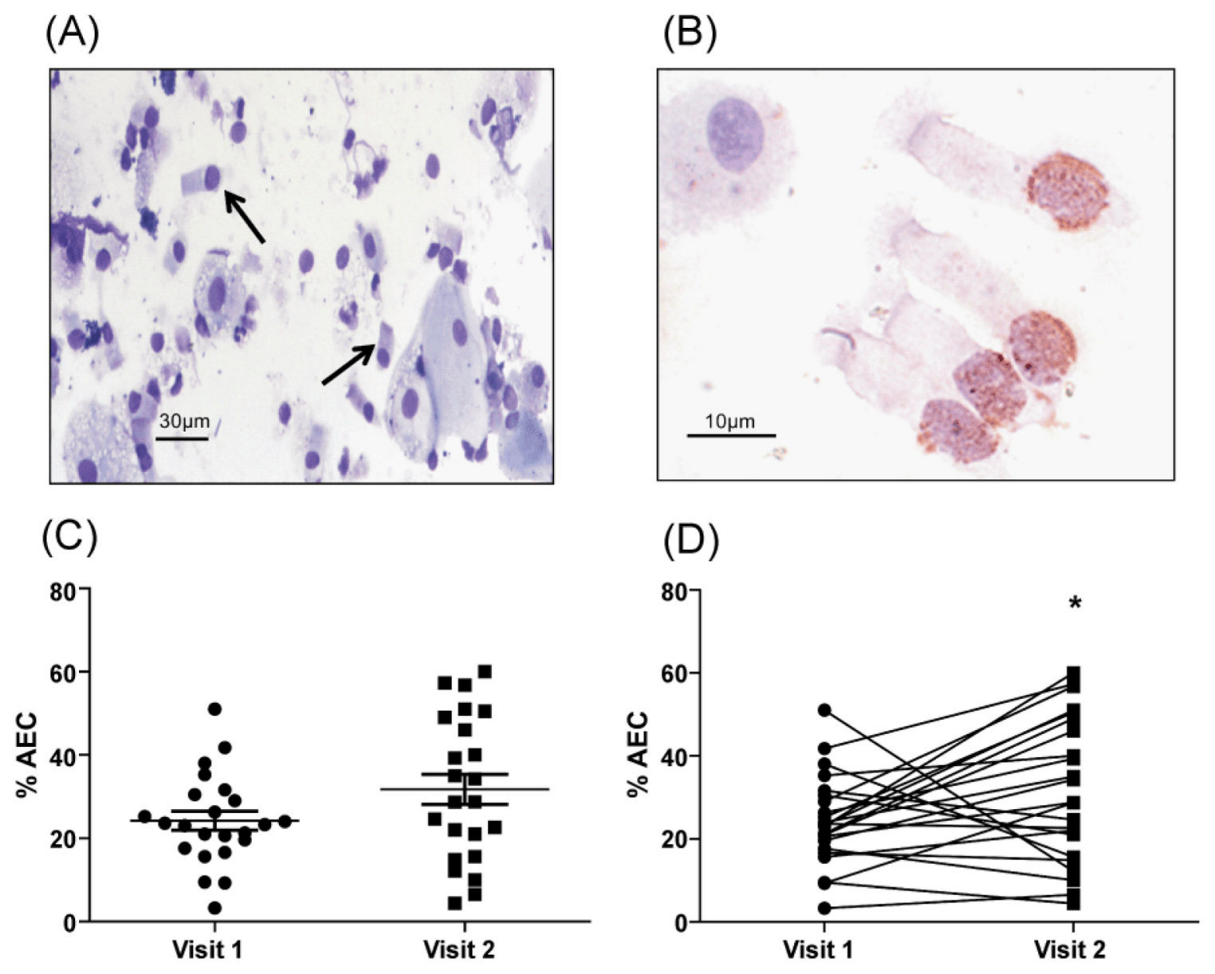
Airway epithelial cell (AEC) shedding into induced sputum is increased after fluticasone exposure. (A) Representative image of Geimsa stained cytospin demonstrated epithelial cell shedding (arrows indicate bronchial epithelial cells); (B) Representative image of TUNEL staining of shed AEC; (C,D) Difference in the % AEC of total cells counted in the sputum analysis, mean values (C) and paired values (D) at visit 1 and visit 2. (*p<0.05)

The percentage of AEC in induced sputum increased from 24.2 (SEM ±2.3) to 31.8 (SEM ±3.6) following fluticasone treatment. There was no significant difference in the means (p=0.08) (Figure 2C) however the difference by paired analysis was significant at p<0.05 (Figure 2D). All of the shed AECs in our study were apoptotic, as determined by terminal deoxynucleotidyl transferase dUTP nick end labeling (TUNEL) staining (Figure 2A and 2B).

### DTPA clearance

**Figure 3:**
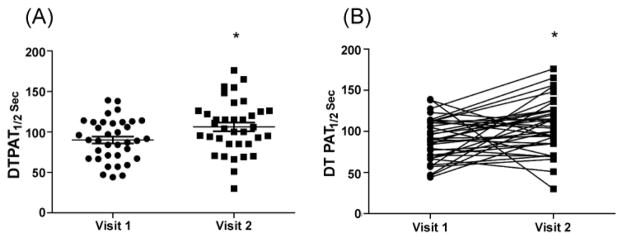
Diethylenetriaminepentacetic acid (DTPA) clearance is reduced after exposure to inhaled fluticasone. Subjects inhaled Technetium-labeled diethylenetriaminepentacetic acid (99mTc-DTPA) at visit 1 and 2 and images were recorded every 30 seconds for a total period of 30 minutes. Analysis done on those scans to calculate T1/2 value at visit 1 and visit 2 (*p<0.05)

The mean (SEM) half-life of DTPA in the lungs increased from 90.1 (4.3) seconds to 106 (5.4) seconds following 28 days of inhaled fluticasone. The difference between the means was significant at p<0.05 (Figure 3A), and paired analysis showed a significant increase in T½ between visit 1 and visit 2 at p<0.02 (Figure 3B).

### Albumin influx

Albumin levels in induced sputum decreased from 761 (SEM ±72) μg/ml to 618 (SEM ±64) μg/ml following 4 weeks of inhaled fluticasone.

**Figure 4:**
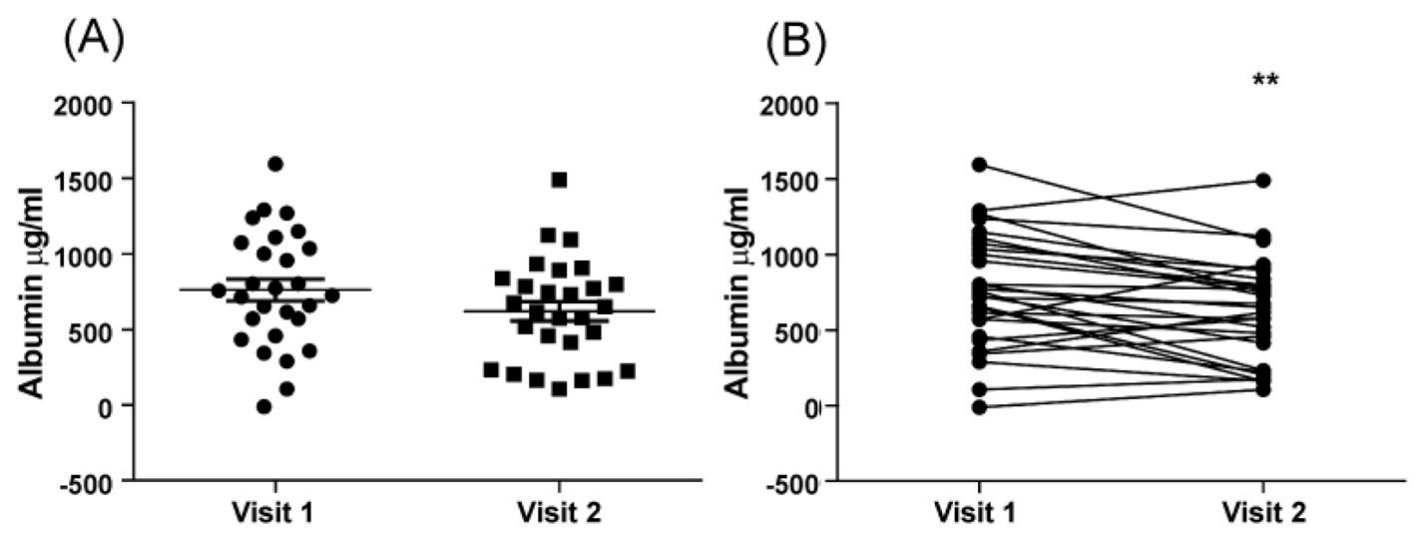
Albumin levels in induced sputum are reduced following exposure to inhaled fluticasone. ELISA was performed on unprocessed sputum samples to detect albumin levels at visit 1 and visit 2. Data is represented as mean value (A) and paired value (B) (**p<0.005)

There was no significant difference between the means p=0.14 (Figure 4A) however paired analysis showed a highly significant reduction in albumin levels following fluticasone treatment at p<0.005 (Figure 4B).

### Airway inflammation

There was no significant difference in the levels of pro-inflammatory cytokines IL-6 and IL-8 following fluticasone treatment (Table 1). HB-EGF levels were similarly unchanged. There was no difference in the inflammatory differential cell counts in the induced sputum following fluticasone exposure (data not shown).

Cell free supernatants from induced sputum were analyzed for IL-6, IL-8 and HB-EGF by ELISA (as described in Material and Methods). No change was observed for IL-6, IL-8 and HB-EGF after fluticasone exposure. Data are mean (SEM). P- Value refers to paired t-test.

## Discussion

The airway epithelium is constantly exposed to inhaled agents that injure the epithelium, and maintains homeostasis through an efficient and highly regulated cycle of injury, inflammation and repair [34]. Our results demonstrate that subjects taking regular inhaled fluticasone for four weeks manifest a degree of airway epithelial injury reflected by a significant increase in the number of shed apoptotic AECs. Epithelial injury occurred in the absence of measurable inflammatory response, and was associated with a reduction in airway permeability and increased barrier function, likely representing an active repair response.

Epithelial shedding and desquamation is considered by many to be a hallmark of asthma, based on post-mortem and biopsy studies [7,9,35,36], along with findings of increased numbers of epithelial cells in sputum [37] and bronchoalveolar lavage fluid [38]. Epithelial loss is largely confined to the columnar epithelial cells [39], and is accompanied by phenotypic changes in the epithelium consistent with the repair process. Persistent repair mechanisms are also implicated in airway remodeling [40,41]. These include upregulation of cyclin-dependent kinase inhibitor, p21 (waf) [42], EGFR [43] and CD-44 [44,45]. In asthma, epithelial shedding is largely attributed to the induction of apoptosis in AECs by infiltrating T cells and eosinophils along with increased pro-inflammatory cytokines including IFN-gamma and TNF-alpha [46]. While cell-mediated inflammation is reduced by corticosteroids, our study was performed in normal individuals without baseline airway inflammation and we observed no changes in the inflammatory indices in induced sputum following corticosteroid therapy. Therefore the epithelial shedding observed in this study likely represents direct apoptotic effect of corticosteroids on AECs as previously demonstrated *in vitro* and in animal studies by ours [23–25,30] and other groups [47].

The potential significance of this corticosteroid induced AEC shedding in asthma is unclear. It might be expected that in asthma, any direct cytopathic effects of corticosteroid would be offset by the beneficial anti-inflammatory effects. We have previously shown however that pre-treatment with corticosteroid reduced allergen-induced inflammation but did not reduce epithelial shedding in an animal model of allergic asthma [23]. Direct corticosteroid-induced AEC injury may be a factor in airway remodeling and explain at least in part the failure of steroid therapy to impact on this process. However, the response of asthmatic epithelial cells to corticosteroids may be different to that of phenotypically normal cells. Vignola and colleagues previously demonstrated increased levels of the anti-apoptotic molecule Bcl-2 in asthmatic subjects compared to controls and there was no difference in the number of apoptotic epithelial cells between corticosteroid-treated and -untreated asthmatic subjects in their study of bronchial biopsies [40]. It would be important therefore to define these effects in subjects with asthma.

The airway mucosa exhibits properties of a selective and tightly regulated permeability barrier that is direction dependent [48], properties which have been used experimentally to assess the presence of airway damage in response to various epithelial insults. Clearance of inhaled labeled-DTPA is considered to be an accurate measurement of lung epithelial permeability, as movement of this molecule into the circulation is limited by the epithelium rather than the endothelium [31]. DTPA is a small hydrophilic molecule that is believed to be cleared from the airway in an apical to basolateral direction via paracellular diffusion; this diffusion is increased following exposure to an allergen or other inflammatory insult [12,49–51] and is thought to be mediated by alterations in cellular contacts such as tight junctions and adherents junctions [12,49]. In addition to DTPA clearance, the presence of serum proteins such as albumin in the airway and measured in induced sputum may reflect altered barrier function in the basolateral-to-apical direction. Plasma extravasation into the sub-epithelial tissue occurs rapidly in response to inhaled allergen reflecting an acute vascular response, but extravasation into the epithelium is not observed [52]. The sustained transepithelial exudation of plasma seen in chronic airway inflammation reflects epithelial injury and repair [53], as almost all (92%) of resistance to albumin flux across the epithelial-capillary barrier occurs in the epithelium [54]. Passive bulk flow of plasma through a “leaky” epithelium is regulated by cellular junctions [48,55], or via diffusive vesicular transport across cells [54].

Intuitively one might expect that epithelial shedding would result in increased permeability and loss of barrier function. However, the complex structure and repair characteristics of the airway epithelium are such that this is not the case. *In vivo* experiments in guinea-pig trachea demonstrate that epithelial shedding is immediately followed by repair responses directed towards covering of the basement membrane and epithelial restitution [52,53,56]. The microcirculation exudes a plasma-rich fibrin-fibronectin gel rich in inflammatory cells, while the cells neighboring the denuded area dedifferentiate, flatten and migrate over the membrane to fill the gap. Columnar cells are likely more easily shed than basal cells [57], and the residual basal cells undergo extensive flattening and develop interdigitating cytoplasmic protrusions observed by electron microscopy [34]. The large flattened cells again have less junctional area than the shed columnar cells, with less potential for paracellular transit of proteins. Thus, in the setting of normal repair, desquamative processes may be accompanied by the apparent paradox of reduced permeability, as a mechanism to reduce further injury [48]. The observation of reduced clearance of DTPA and decreased luminal albumin in the context of epithelial shedding in our normal subjects likely represents initiation of a normal injury repair response.

Corticosteroids have long been known to reduce endothelial permeability, particularly at the blood brain barrier, where they are used therapeutically to reduce cerebral edema [58]. The mechanisms of improved barrier function are not fully understood, but include glucocorticoid mediated induction of junctional proteins including occludin, ZO-1 and claudin [59–61]. Evidence from lung epithelium is less abundant, but suggests similar effects. In a co-culture model of immortalized lung epithelial cell lines in monolayer with primary human pulmonary microvascular endothelial cells, dexamethasone treatment resulted in increased membrane staining of E-Cadherin and ZO-1 in the NCI H441 epithelial cells which was associated with increased transepithelial resistance (TER), a surrogate of reduced permeability [62]. While it is difficult to extrapolate these results to the polarized pseudo-stratified airway epithelium *in vivo*, it is possible that modulation of junctional proteins contributes to the observed reduction in permeability.

Some studies of epithelial permeability have failed to demonstrate a significant difference between normal subjects and mild stable asthmatics [63–65]. Lemarchand *et al.* [66] however showed increased DTPA clearance in asthmatics during acute exacerbations, which decreased within 4 weeks of the acute attack but still remained increased compared to the control group. Ilowite *et al.* [67] adjusted their results for mucociliary clearance, which was reduced in the asthmatics, and found that while total clearance was the same between asthmatics and controls, airway permeability was in fact increased in the asthmatics [50]. Another recent study of chronic persistent asthma, which excluded the component of mucociliary transport by careful exclusion of the hilar regions, found increased permeability in asthma compared to control and the degree of permeability was correlated with severity of asthma [68]. It would appear therefore that epithelial damage in asthma, at least during acute attacks, is associated with loss of barrier function.

This study was performed in normal subjects in order to isolate the cytopathic effects of corticosteroids on the epithelium from the anti-inflammatory effects. One limitation of this approach is in then extrapolating the results to the asthmatic airway, which by definition will have differences in the injury and repair responses. Asthma is characterized by a cycle of chronic injury and dysregulated repair [69,70]. Recent studies demonstrate that airway epithelial cells from asthmatic children express significantly less fibronectin compared to non-asthmatic controls, resulting in reduced reparative capacity [71]. Thus, while corticosteroid induced cytopathology and epithelial injury may be inconsequential in the context of normal repair mechanisms which quickly restore and even improve barrier function, where these responses are inadequate or abnormal, permeability may increase. The first studies of inhaled corticosteroids and permeability in asthma used low daily doses (200 μg and 400 μg Beclomethasone) for short duration (1 week), and found no difference in DTPA clearance rates [72,73]. However, in the only other human study of the effect of chronic inhaled corticosteroids on airway permeability, treatment with corticosteroids (budesonide) in atopic asthmatic children did in fact result in increased permeability as measured by DTPA clearance [74].

Evidence from both biopsy and differentiated culture studies indicate that expression and assembly of tight junctions is abnormal in asthma [75,76]. This may contribute to the epithelial “fragility” in asthma, while failure to upregulate tight junctions in response to corticosteroid may contribute to an altered permeability response in the context of epithelial shedding. It is unclear whether the direct cytopathic effects of corticosteroids observed in this study are glucocorticoid receptor-mediated and would occur in the population of steroid resistant asthmatics. Certainly these effects in the absence of the beneficial anti-inflammatory effects could be potentially catastrophic with regard to airways remodeling. This further underlines the importance of early identification of these patients and avoiding the use of both systemic and inhaled corticosteroids.

The Flovent inhaler used in this study consists of 99.66 – 99.91% 1,1,1,2-tetrafluoroethane propellant (carrier) and 0.09 – 0.34% fluticasone propionate powder. 1,1,1,2-tetrafluoroethane (also known as the Glaxo compound HFA134a) was developed in the early 1990s as an alternative propellant to chlorofluorocarbons as it has a far lower ozone-depleting potential. Inhaled radiolabelled HFA134a is rapidly removed from the body by ventilation and does not accumulate significantly in any specific region of the body [77]. Multiple other safety studies have demonstrated that HFA134a has no negative impact on various respiratory and systemic health indices in healthy volunteers [78,79] and in patients with mild-moderate asthma [80–82]. Even whole body exposure to increasing concentrations of HFA234a did not trigger any adverse effects, including upper respiratory tract irritation [83]. There has been a single case of extrinsic allergic alveolitis with eosinophil infiltration triggered by 1,1,1,2-tetrafluoroethane inhalation [84], but the vast majority of individuals do not show any adverse health effects. There is little evidence to suggest that the effects we observed in patients using the Flovent inhaler were caused by the propellant.

## Conclusions

In summary, our results demonstrate that normal subjects taking inhaled fluticasone at clinically relevant doses for 4 weeks show evidence of epithelial damage in the absence of airway inflammation. This is associated with reduced airway permeability and albumin extravasation, reflecting repair. The airway epithelium in asthma is held in a cycle of chronic injury, inflammation and dysregulated repair. Inhaled corticosteroids in asthma may contribute to epithelial injury, while at the same time reducing the accompanying inflammatory response. Failure of appropriate repair mechanisms may result in increased permeability and increased susceptibility to inhalational injury. We have no reason to believe that the observed results are unique to the particular steroid used in this study; however further studies using other inhaled steroid preparations and carrier only placebo controls would be of interest.

While the beneficial effects of corticosteroids on cell-mediated immunopathology in asthma are manifold, our study suggests that the direct toxic effects on the epithelium may not be inconsequential, particularly in the context of inappropriate repair, and could in fact contribute to airway remodeling. Care should be taken in extrapolating our findings to asthmatic subjects recognizing the potential differences in apoptotic susceptibility, inflammation and repair. Given the widespread use of inhaled corticosteroids and their failure to prevent airway remodeling, further careful investigation of these effects in asthmatic subjects is warranted.

## Figures and Tables

**Table 1:** Concentrations of inflammatory mediators (IL-6, IL-8 and HB-EGF) in induced sputum

Concentration (pg/ml)	Visit 1	Visit 2	P-value
IL-6	113.9 (139.4)	79.7 (85.9)	0.27
IL-8	755.3 (906.1)	611.8 (757.2)	0.72
HB-EGF	609.6 (298.1)	679.8 (457.2)	0.54

## References

[1] Becker A, Lemière C, Bérubé D, Boulet LP, Ducharme FM (2005). Summary of recommendations from the Canadian Asthma Consensus guidelines, 2003. CMAJ.

[2] Cohn L, Elias JA, Chupp GL (2004). Asthma: mechanisms of disease persistence and progression. Annu Rev Immunol.

[3] Fahy JV (2001). Remodeling of the airway epithelium in asthma. Am J Respir Crit Care Med.

[4] Murphy DM, O’Byrne PM (2010). Recent advances in the pathophysiology of asthma. Chest.

[5] Holgate ST, Roberts G, Arshad HS, Howarth PH, Davies DE (2009). The role of the airway epithelium and its interaction with environmental factors in asthma pathogenesis. Proc Am Thorac Soc.

[6] Swindle EJ, Collins JE, Davies DE (2009). Breakdown in epithelial barrier function in patients with asthma: identification of novel therapeutic approaches. J Allergy Clin Immunol.

[7] Jeffery PK, Wardlaw AJ, Nelson FC, Collins JV, Kay AB (1989). Bronchial biopsies in asthma. An ultrastructural, quantitative study and correlation with hyperreactivity. Am Rev Respir Dis.

[8] Vignola AM, Chanez P, Campbell AM, Souques F, Lebel B (1998). Airway inflammation in mild intermittent and in persistent asthma. Am J Respir Crit Care Med.

[9] Laitinen LA, Heino M, Laitinen A, Kava T, Haahtela T (1985). Damage of the airway epithelium and bronchial reactivity in patients with asthma. Am Rev Respir Dis.

[10] Barbato A, Turato G, Baraldo S, Bazzan E, Calabrese F (2006). Epithelial damage and angiogenesis in the airways of children with asthma. Am J RespirCrit Care Med.

[11] Erjefält JS (2010). The airway epithelium as regulator of inflammation patterns in asthma. ClinRespir J.

[12] Holgate ST (2008). Pathogenesis of asthma. Clin Exp Allergy.

[13] Bateman ED, Hurd SS, Barnes PJ, Bousquet J, Drazen JM (2008). Global strategy for asthma management and prevention: GINA executive summary. Eur Respir J.

[14] Barnes PJ (2000). Molecular basis for corticosteroid action in asthma. Chem Immunol.

[15] LeVan TD, Babin EA, Yamamura HI, Bloom JW (1999). Pharmacological characterization of glucocorticoid receptors in primary human bronchial epithelial cells. Biochem Pharmacol.

[16] Stellato C (2007). Glucocorticoid actions on airway epithelial responses in immunity: functional outcomes and molecular targets. J Allergy Clin Immunol.

[17] Barnes N (1998). Relative safety and efficacy of inhaled corticosteroids. J Allergy Clin Immunol.

[18] Calverley PM (2004). Effect of corticosteroids on exacerbations of asthma and chronic obstructive pulmonary disease. Proc Am Thorac Soc.

[19] Murray CS (2008). Can inhaled corticosteroids influence the natural history of asthma?. Curr Opin Allergy Clin Immunol.

[20] Bisgaard H, Bønnelykke K (2010). Long-term studies of the natural history of asthma in childhood. J Allergy Clin Immunol.

[21] Goleva E, Hauk PJ, Boguniewicz J, Martin RJ, Leung DY (2007). Airway remodeling and lack of bronchodilator response in steroid-resistant asthma. J Allergy Clin Immunol.

[22] Sumi Y, Hamid Q (2007). Airway remodeling in asthma. Allergol Int.

[23] Dorscheid DR, Low E, Conforti A, Shifrin S, Sperling AI (2003). Corticosteroid-induced apoptosis in mouse airway epithelium: effect in normal airways and after allergen-induced airway inflammation. J Allergy Clin Immunol.

[24] Dorscheid DR, Patchell BJ, Estrada O, Marroquin B, Tse R (2006). Effects of corticosteroid-induced apoptosis on airway epithelial wound closure in vitro. Am J Physiol Lung Cell Mol Physiol.

[25] Dorscheid DR, Wojcik KR, Sun S, Marroquin B, White SR (2001). Apoptosis of airway epithelial cells induced by corticosteroids. Am J Respir Crit Care Med.

[26] MacRedmond RE, Greene CM, Dorscheid DR, McElvaney NG, O’Neill SJ (2007). Epithelial expression of TLR4 is modulated in COPD and by steroids, salmeterol and cigarette smoke. Respir Res.

[27] Singhera GK, Chan TS, Cheng JY, Vitalis TZ, Hamann KJ (2006). Apoptosis of viral-infected airway epithelial cells limit viral production and is altered by corticosteroid exposure. Respir Res.

[28] Tse R, Marroquin BA, Dorscheid DR, White SR (2003). Beta-adrenergic agonists inhibit corticosteroid-induced apoptosis of airway epithelial cells. Am J Physiol Lung Cell Mol Physiol.

[29] Undevia NS, Dorscheid DR, Marroquin BA, Gugliotta WL, Tse R (2004). Smad and p38-MAPK signaling mediates apoptotic effects of transforming growth factor-beta1 in human airway epithelial cells. Am J Physiol Lung Cell Mol Physiol.

[30] White SR, Dorscheid DR (2002). Corticosteroid-induced apoptosis of airway epithelium: a potential mechanism for chronic airway epithelial damage in asthma. Chest.

[31] Effros RM, Mason GR (1983). Measurements of pulmonary epithelial permeability in vivo. Am Rev Respir Dis.

[32] Levin S (1984). Statistical methods, Textbook of Nuclear Medicine.

[33] Allahverdian S, Harada N, Singhera GK, Knight DA, Dorscheid DR (2008). Secretion of IL-13 by airway epithelial cells enhances epithelial repair via HB-EGF. Am J Respir Cell Mol Biol.

[34] Erjefält JS, Sundler F, Persson CG (1997). Epithelial barrier formation by airway basal cells. Thorax.

[35] Dunnil MS (1960). The pathology of asthma, with special reference to changes in the bronchial mucosa. J Clin Pathol.

[36] Houston JC, De Navasquez S, Trounce JR (1953). A clinical and pathological study of fatal cases of status asthmaticus. Thorax.

[37] Naylor B (1962). The shedding of the mucosa of the bronchial tree in asthma. Thorax.

[38] Wardlaw AJ, Dunnette S, Gleich GJ, Collins JV, Kay AB (1988). Eosinophils and mast cells in bronchoalveolar lavage in subjects with mild asthma. Relationship to bronchial hyperreactivity. Am Rev Respir Dis.

[39] Beasley R, Roche WR, Roberts JA, Holgate ST (1989). Cellular events in the bronchi in mild asthma and after bronchial provocation. Am Rev Respir Dis.

[40] Vignola AM, Chiappara G, Siena L, Bruno A, Gagliardo R (2001). Proliferation and activation of bronchial epithelial cells in corticosteroid-dependent asthma. J Allergy Clin Immunol.

[41] Cohen L, EX, Tarsi J, Ramkumar T, Horiuchi TK (2007). Epithelial cell proliferation contributes to airway remodeling in severe asthma. Am J RespirCrit Care Med.

[42] Puddicombe SM, Torres-Lozano C, Richter A, Bucchieri F, Lordan JL (2003). Increased expression of p21(waf) cyclin-dependent kinase inhibitor in asthmatic bronchial epithelium. Am J Respir Cell Mol Biol.

[43] Puddicombe SM, Polosa R, Richter A, Krishna MT, Howarth PH (2000). Involvement of the epidermal growth factor receptor in epithelial repair in asthma. FASEB J.

[44] Lackie PM, Baker JE, Günthert U, Holgate ST (1997). Expression of CD44 isoforms is increased in the airway epithelium of asthmatic subjects. Am J Respir Cell Mol Biol.

[45] Peroni DG, Djukanovic R, Bradding P, Feather IH, Montefort S (1996). Expression of CD44 and integrins in bronchial mucosa of normal and mildly asthmatic subjects. Eur Respir J.

[46] Trautmann A, Kruger K, Akdis M, Muller-Wening D, Akkaya A (2005). Apoptosis and loss of adhesion of bronchial epithelial cells in asthma. Int Arch Allergy Immunol.

[47] Andersson K, Shebani EB, Makeeva N, Roomans GM, Servetnyk Z (2010). Corticosteroids and montelukast: effects on airway epithelial and human umbilical vein endothelial cells. Lung.

[48] Persson CG, Andersson M, Greiff L, Svensson C, Erjefält JS (1995). Airway permeability. Clin Exp Allergy.

[49] Knight D (2002). Increased permeability of asthmatic epithelial cells to pollutants. Does this mean that they are intrinsically abnormal?. Clin Exp Allergy.

[50] Ilowite JS, Bennett WD, Sheetz MS, Groth ML, Nierman DM (1989). Permeability of the bronchial mucosa to 99mTc-DTPA in asthma. Am Rev Respir Dis.

[51] Kennedy SM, Elwood RK, Wiggs BJ, Paré PD, Hogg JC (1984). Increased airway mucosal permeability of smokers. Relationship to airway reactivity. Am Rev Respir Dis.

[52] Erjefält JS, Andersson P, Gustafsson B, Korsgren M, Sonmark B (1998). Allergen challenge-induced extravasation of plasma in mouse airways. Clin Exp Allergy.

[53] Erjefält JS, Sundler F, Persson CG (1996). Eosinophils, neutrophils, and venular gaps in the airway mucosa at epithelial removal-restitution. Am J Respir Crit Care Med.

[54] Gorin AB, Stewart PA (1979). Differential permeability of endothelial and epithelial barriers to albumin flux. J Appl Physiol Respir Environ Exerc Physiol.

[55] Persson CG (1990). Plasma exudation in tracheobronchial and nasal airways: a mucosal defence mechanism becomes pathogenic in asthma and rhinitis. Eur Respir J Suppl.

[56] Erjefält I, Greiff L, Persson CG (1993). Exudation versus absorption across the airway epithelium. Pharmacol Toxicol.

[57] Montefort S, Roberts JA, Beasley R, Holgate ST, Roche WR (1992). The site of disruption of the bronchial epithelium in asthmatic and non-asthmatic subjects. Thorax.

[58] Rabinstein AA (2006). Treatment of cerebral edema. Neurologist.

[59] Förster C, Waschke J, Burek M, Leers J, Drenckhahn D (2006). Glucocorticoid effects on mouse microvascular endothelial barrier permeability are brain specific. J Physiol.

[60] Romero IA, Radewicz K, Jubin E, Michel CC, Greenwood J (2003). Changes in cytoskeletal and tight junctional proteins correlate with decreased permeability induced by dexamethasone in cultured rat brain endothelial cells. Neurosci Lett.

[61] Kröll S, El-Gindi J, Thanabalasundaram G, Panpumthong P, Schrot S (2009). Control of the blood-brain barrier by glucocorticoids and the cells of the neurovascular unit. Ann N Y Acad Sci.

[62] Hermanns MI, Unger RE, Kehe K, Peters K, Kirkpatrick CJ (2004). Lung epithelial cell lines in coculture with human pulmonary microvascular endothelial cells: development of an alveolo-capillary barrier in vitro. Lab Invest.

[63] Del Donno M, Chetta A, Foresi A, Gavaruzzi G, Ugolotti G (1997). Lung epithelial permeability and bronchial responsiveness in subjects with stable asthma. Chest.

[64] Elwood RK, Kennedy S, Belzberg A, Hogg JC, Paré PD (1983). Respiratory mucosal permeability in asthma. Am Rev Respir Dis.

[65] O’Byrne PM, Dolovich M, Dirks R, Roberts RS, Newhouse MT (1984). Lung epithelial permeability: relation to nonspecific airway responsiveness. J Appl Physiol Respir Environ Exerc Physiol.

[66] Lemarchand P, Chinet T, Collignon MA, Urzua G, Barritault L (1992). Bronchial clearance of DTPA is increased in acute asthma but not in chronic asthma. Am Rev Respir Dis.

[67] Bennett WD, Ilowite JS (1989). Dual pathway clearance of 99mTc-DTPA from the bronchial mucosa. Am Rev Respir Dis.

[68] Bhure UN, Bhure SU, Bhatt BM, Mistry S, Pednekar SJ (2009). Lung epithelial permeability and inhaled furosemide: added dimensions in asthmatics. Ann Nucl Med.

[69] Bucchieri F, Puddicombe SM, Lordan JL, Richter A, Buchanan D (2002). Asthmatic bronchial epithelium is more susceptible to oxidant-induced apoptosis. Am J Respir Cell Mol Biol.

[70] Holgate ST (2008). The airway epithelium is central to the pathogenesis of asthma. Allergol Int.

[71] Kicic A, Hallstrand TS, Sutanto EN, Stevens PT, Kobor MS (2010). Decreased fibronectin production significantly contributes to dysregulated repair of asthmatic epithelium. Am J Respir Crit Care Med.

[72] Wang SJ, Kao CH, Lin WY, Hsu CY, Chang CP (1995). Effects of inhalation of steroids on lung permeability in patients with asthma. Clin Nucl Med.

[73] Tsai SC, Kao CH, Wang SJ, Lan JL, ChangLai SP (1995). Effects of corticosteroid inhalation therapy on lung ventilation and alveolar permeability in asthma using TC-99M DTPA radioaerosol inhalation lung scintigraphy. Gaoxiong Yi Xue Ke Xue ZaZhi.

[74] Yuksel H, Yuksel D, Demir E, Tanaç R (2001). Influence of inhaled steroids on pulmonary epithelial permeability to Tc99m-dTPA in atopic asthmatic children. J Investig Allergol Clin Immunol.

[75] de Boer WI, Sharma HS, Baelemans SM, Hoogsteden HC, Lambrecht BN (2008). Altered expression of epithelial junctional proteins in atopic asthma: possible role in inflammation. Can J Physiol Pharmacol.

[76] Wan H, Winton HL, Soeller C, Gruenert DC, Thompson PJ (2000). Quantitative structural and biochemical analyses of tight junction dynamics following exposure of epithelial cells to house dust mite allergen Der p 1. Clin Exp Allergy.

[77] Pike VW, Aigbirhio FI, Freemantle CA, Page BC, Rhodes CG (1995). Disposition of inhaled 1,1,1,2-tetrafluoroethane (HFA134A) in healthy subjects and in patients with chronic airflow limitation. Measurement by 18F-labeling and whole-body gamma-counting. Drug Metab Dispos.

[78] Donnell D, Harrison LI, Ward S, Klinger NM, Ekholm BP (1995). Acute safety of the CFC-free propellant HFA-134a from a pressurized metered dose inhaler. Eur J Clin Pharmacol.

[79] Kirby SM, Smith J, Ventresca GP (1995). Salmeterol inhaler using a non-chlorinated propellant, HFA134a: systemic pharmacodynamic activity in healthy volunteers. Thorax.

[80] Jenkins M (1995). Clinical evaluation of CFC-free metered dose inhalers. J Aerosol Med.

[81] Ramsdell JW, Colice GL, Ekholm BP, Klinger NM (1998). Cumulative dose response study comparing HFA-134a albuterol sulfate and conventional CFC albuterol in patients with asthma. Ann Allergy Asthma Immunol.

[82] Nelson HS, Kane RE, Petillo J, Banerji D (2000). Long-term safety of a non-chlorofluorocarbon-containing triamcinolone acetonide inhalation aerosol in patients with asthma. Azmacort HFA Study Group. J Asthma.

[83] Emmen HH, Hoogendijk EM, Klöpping-Ketelaars WA, Muijser H, Duistermaat E (2000). Human safety and pharmacokinetics of the CFC alternative propellants HFC 134a (1,1,1,2-tetrafluoroethane) and HFC 227 (1,1,1,2,3,3, 3-heptafluoropropane) following whole-body exposure. Regul Toxicol Pharmacol.

[84] Ishiguro T, Yasui M, Nakade Y, Kimura H, Katayama N (2007). Extrinsic allergic alveolitis with eosinophil infiltration induced by 1,1,1,2-tetrafluoroethane (HFC-134a): a case report. Intern Med.

